# Stick–slip behaviour on Au(111) with adsorption of copper and sulfate

**DOI:** 10.3762/bjnano.6.85

**Published:** 2015-03-26

**Authors:** Nikolay Podgaynyy, Sabine Wezisla, Christoph Molls, Shahid Iqbal, Helmut Baltruschat

**Affiliations:** 1 Institute of Physical and Theoretical Chemistry, University of Bonn, Roemerstrasse 164, D-53117 Bonn, Germany

**Keywords:** AFM, friction, friction force microscopy, nanotribology, underpotential deposition

## Abstract

Several transitions in the friction coefficient with increasing load are found on Au(111) in sulfuric acid electrolyte containing Cu ions when a monolayer (or submonolayer) of Cu is adsorbed. At the corresponding normal loads, a transition to double or multiple slips in stick–slip friction is observed. The stick length in this case corresponds to multiples of the lattice distance of the adsorbed sulfate, which is adsorbed in a √3 × √7 superstructure on the copper monolayer. Stick–slip behaviour for the copper monolayer as well as for 2/3 coverage can be observed at *F*_N_ ≥ 15 nN. At this normal load, a change from a small to a large friction coefficient occurs. This leads to the interpretation that the tip penetrates the electrochemical double layer at this point. At the potential (or point) of zero charge (pzc), stick–slip resolution persists at all normal forces investigated.

## Introduction

Atomic-scale friction processes constitute a fascinating field of research which has been opened by the invention of the atomic force microscope (AFM) [1]. The AFM allows us to determine the force necessary to move a cantilever tip laterally across the surface with atomic resolution. A theoretical model describing this so-called stick–slip motion was provided by Tomlinson [2]. Tip atoms in contact with the surface remain at a certain surface position with a minimum of potential energy until the increasing lateral force initiates a slip toward the next potential minimum. Many interesting aspects on the origin of friction and the underlying dissipative processes have been elucidated so far, but an overall understanding is still far from being complete [3–6].

Investigations at surfaces performed under electrochemical conditions offer some advantages compared to those performed in air or under ultra high vacuum (UHV) conditions: simply by varying the potential of the working electrode, the electrode surface can quickly and reversibly be modified by adsorption of a foreign metal or other substances, while the degree of unwanted contamination can be kept as low as under UHV conditions.

There are only a few older publications reporting on AFM friction studies on HOPG (highly oriented pyrolytic graphite) and polycristalline Ag under electrochemical conditions, examining the dependence of friction on potential and the adsorption of anions [7–9]. Our group recently started to study friction forces on single crystal electrodes under electrochemical conditions. In [10–11] we investigated the effect of copper under potential deposition (UPD) on Au(111) and Pt(111) on friction and found an increase in friction force after adsorption of a sub- or monolayer of copper. A particularly high friction was observed at the potential where the 2/3 Cu adlayer was formed. This was later corroborated by Bennewitz and coworkers [12]. A transition in friction coefficient was found at a certain normal load on a copper monolayer which was ascribed to a normal load dependent penetration of the double layer or even displacements of adsorbates [10,13]. We showed that the pressure necessary for the displacement of adsorbates such as a UPD metal calculated from a typical pressure dependence of the adsorption free enthalpy (as given by the adsorption volume, cf. [14–15]) does correspond to the pressure exerted by the tip. The friction force on UPD copper in presence of chloride is much smaller than in sulfuric acid solution. Upon the adsorption of sulfate ions on Au(111), we observed a considerable increase in friction force [10,13].

Bennewitz, Hausen and Gosvami showed that stick–slip resolution can be obtained for this adlayer [12] Labuda et al. [16] found that atomic resolution on gold is most easily achieved at the potential of zero charge (pzc). They observed "blurred" resolution at higher potential where oxygen starts being adsorbed. Hausen et al. observed a stick–slip periodicity equal to the lattice distance of sulfate anions, which is adsorbed in a √3 × √7 superstructure [17]. They found an increase in friction force upon the adsorption of anions only at normal loads above a certain threshold. As an explanation, the authors consider the squeezing out of water layer when the normal force exceeds a certain threshold [18]. The behaviour of water on crystalline surfaces was described as a viscous structure, which can resist to the tip pressure up to 4 water layers [19]. On the other hand, the properties of viscous water on a gold surface are dependent on the surface potential. For Au(111) in 0.05 M sulfuric acid solution + 1 mM CuSO_4_ the pzc is 0.22 V vs Cu/Cu^2+^ [20]. Labuda et al. [21] obtained atomic resolution of a copper sub-monolayer on gold(111) in perchloric acid solution. They found an increase in friction force after metal adsorption as compared to a clean gold surface.

When the tip is scanning across a monatomic step, friction is largely increased due to the Schwoebel barrier, as has been shown for the NaCl(001)/gas interfaces [22]. We observed the same effect at the Au(111)/electrolyte interfaces [10,13]. Correspondingly, we have shown that friction is higher when the tip is scanning perpendicular to the steps of a stepped Au(665) electrode surface than when scanning parallel; only in the latter case, the effects of Cu UPD on the stepped Au(665) on friction is similar to that on the Au(111) electrode surface [23].

The objective of the present work is a detailed study of the nature of atomic-scale friction with respect to the Au(111) surface, covered with different adsorbates. We would like to find out what factors influence friction and to learn more about the role of the double layer thereupon. It is also our ongoing interest to elucidate whether findings obtained at the solid/gas interface are also valid at the solid electrolyte interface.

In this paper we present the results of investigations of friction forces during UPD and dissolution of Cu/Au(111) and also during sulfate adsorption in sulfuric acid solution. We extend previous measurements to lower loads and also elucidate the conditions for atomic stick–slip. This system has been well studied in a large number of publications and therefore is a model system for fundamental studies. Many different methods have been used including STM [24–25], and also ex situ experiments such as LEED, AES and RHEED [26]. The given results reveal the coverage and the structure of adsorbed copper and sulfate anions on Au(111) and the potential regions at which the different structures can be observed. Gordon et al. [27] found a √3 × √3 copper structure in the 2/3 coverage region using X-ray and QCM methods. Lipkowski et al. [28] used standard electrochemical instrumentation in order to determine the Gibbs excess of copper coadsorbed with SO_4_^2−^ anions.

## Experimental

AFM measurements were performed with a Nanoscope III E controller (Digital Instruments, Santa Barbara, CA) and a commercially available AFM scanner (Molecular Imaging) fitted with an electrochemical cell. The nominal spring constants of the commercial Si cantilevers used (Veeco MPP-31100) were 0.65 N/m. The torsional force constant was determined via Sader's method [29] to be 190 N/m. This method is based on the measurement of the resonant frequency and quality factor of the cantilever. All AFM measurements were performed at room temperature.

Friction force maps shown here are the difference images between both scan directions. Due to the relatively high load used in our experiments, the tip radius quickly approached a value of around 100 nm, but then stayed constant as revealed by SEM images obtained after different usage times. Using the formulae given in [4], the real contact area at a normal load of 15 nN was estimated to around 30 nm^2^ and at 60 nN around 70 nm^2^ [30].

Cyclic voltammograms were recorded with standard electrochemical equipment. The solutions were made of ultrapure H_2_SO_4_ (Merck, suprapure), CuSO_4_ (Merck, >99%, p.a.) and high purity water (millipore, toc *<* 3 ppb, 18*.*2 MΩ·cm). The Au(111) single crystal (obtained from Metal Crystals & Oxides) was oriented with an accuracy of 0.5°. The preparation of the single crystal was performed by flame annealing to red heat for two minutes. The crystal was cooled in argon atmosphere and brought into contact with the deaerated solution at room temperature. All potentials were measured and are quoted with respect to a Cu/Cu^2+^ reference electrode. Cleanliness was controlled by cyclic voltammetry in sulfuric acid.

## Results and Discussion

**Figure 1:**
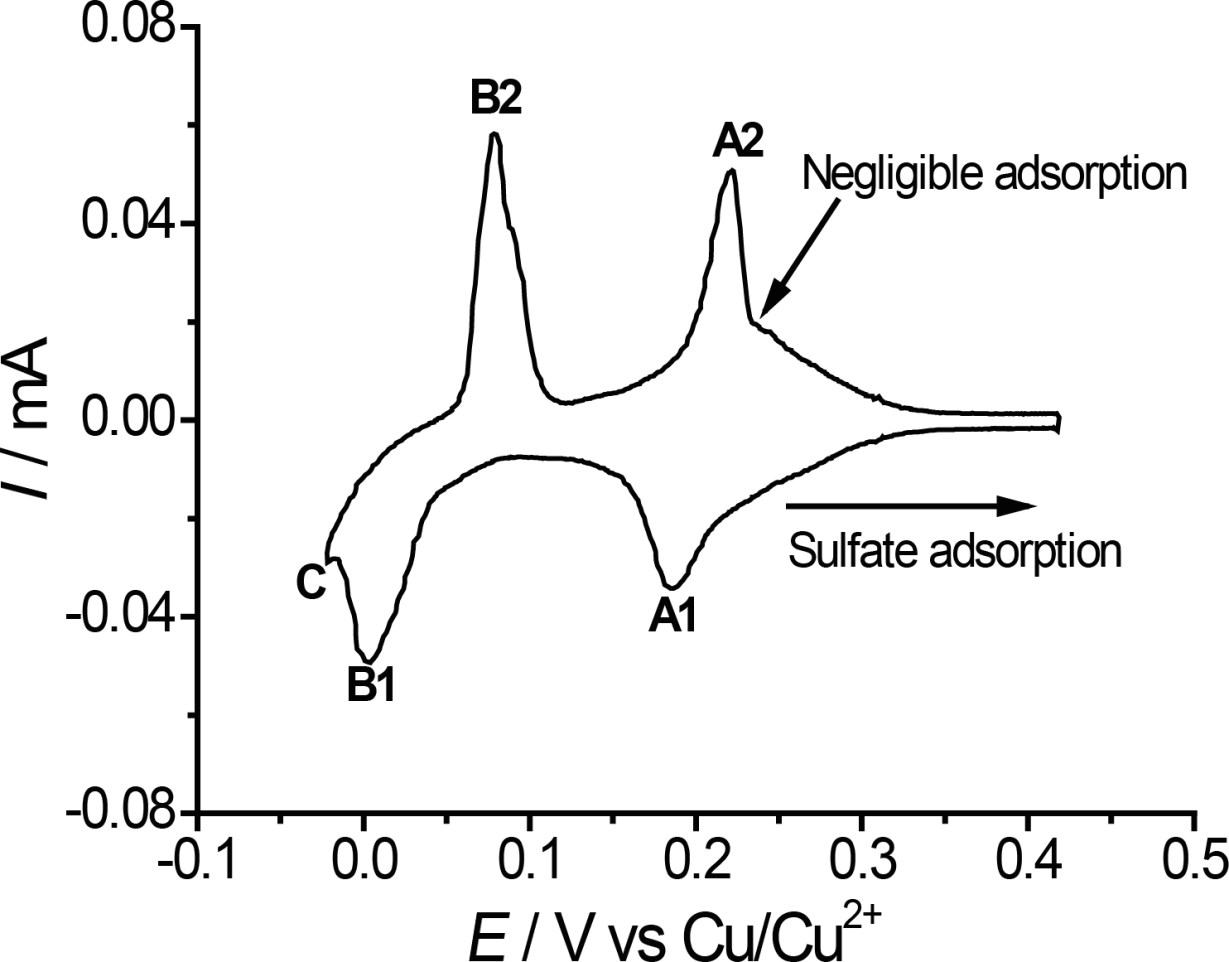
Cyclic voltammogram in 0.05 M H_2_SO_4_ + 4 × 10^−4^ M CuSO_4_. *v* = 50 mV/s.

Figure 1 shows a cyclic voltammogram of Au(111) in 0.05 M H_2_SO_4_ + 4 × 10^−4^ M CuSO_4_ in the AFM cell. The voltammogram can be split into three potential regions (see Figure 1). At potentials *E* ≥ 250 mV only sulfate is adsorbed to the Au(111) surface. The sulfate coverage increases with potential. Between peak A1 and B1, the copper coverage is Θ_Cu_ = 2/3 of a monolayer; copper has a √3 × √3 honeycomb structure. Negative of peaks B and positive of peak C, the latter corresponding to copper bulk deposition, a full monolayer of copper is present. Both the monolayer of copper and the copper 2/3 layer are covered by sulfate anions, adsorbed from solution at potentials *E* ≤ 0.2 V vs Cu/Cu^2+^. On the copper 2/3 layer, sulfate anions occupy the holes in the honeycomb structure, therefore forming a √3 × √3 structure as well. On the copper monolayer, sulfate is adsorbed in a √7 × √3 structure. At the potential of zero charge (pzc) at *E* = 0.22 V, just positive of peak A2 or A1, only a small amount of adsorbate covers the gold surface, according to [28] Θ_Cu_ = 9%; Θ_sulfate_ = 5%.

**Figure 2:**
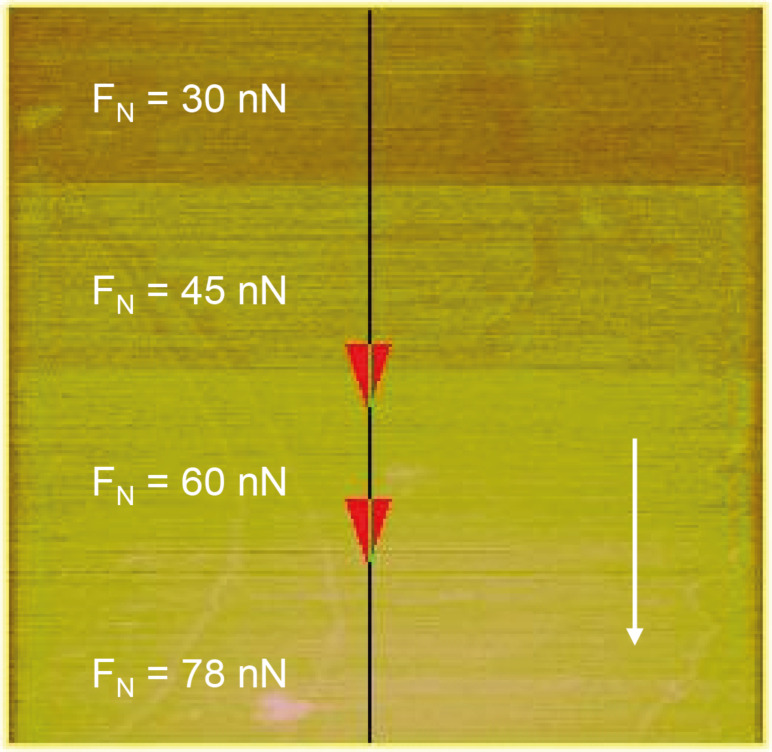
Friction image of Au(111) (in 0.05 M H_2_SO_4_ + 4 × 10^−4^ M CuSO_4_) with a variation of normal load; *E* = 0.22 V (pzc); image size 600 nm × 600 nm.

In Figure 2 the friction image (difference image of trace and retrace signals) of the Au(111) surface at the pzc is presented. The figure also illustrates how the friction force was determined as a function of normal load.

**Figure 3:**
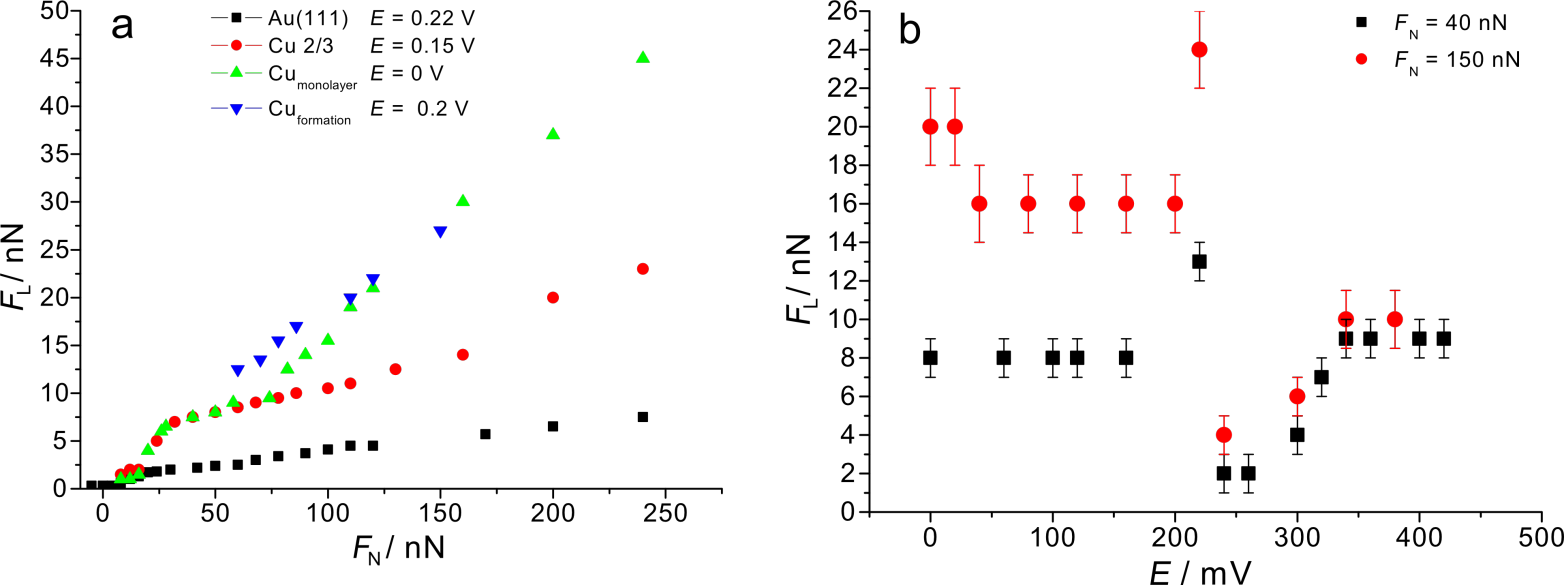
a) Friction force as a function of normal load. Green triangles: *E* = 0 V (Cu monolayer); red circles: *E* = 0.15 V (2/3 Cu coverage); black squares: *E* = 0.22 V (Au pzc). b) Friction force as a function of potential. Black: *F*_N_ = 40 nN; red: *F*_N_ = 150 nN.

The results of these investigations for different potentials are given in Figure 3a,b. Figure 3a shows the dependency of friction force versus normal load for a set of four selected potentials corresponding to different adsorbate structures. As already reported before [11], a transition in the coefficient of friction is observed for a copper monolayer at *F*_N_ = 70 nN. Here, we extended the study to lower normal loads and observed another transition in the friction coefficient at a normal load of *F*_N_ ≈ 15 nN.

The dependency of friction force on potential is shown in Figure 3b. The friction force is independent of potential as long as the adsorbate structure is preserved but changes when the adsorbate structure is changed; it is minimal at the pzc (*E* = 0.22 V). Due to adsorption of sulfate, positive of the pzc, friction force increases with increasing potential, as already shown before for Au(111) in H_2_SO_4_ [13]. The high friction value at *E* ≈ 0.2 V, which exceeds the friction value in the copper 2/3 adlayer region, is constant with time and is found at all normal loads investigated. This potential corresponds to the peak in the cyclic voltammogram which is due to formation of the copper 2/3 layer. At this peak potential, copper makes up about 30% surface coverage. In our previous experiments, such a large friction value was observed when stepping the potential from a potential of zero Cu coverage into the copper 2/3 adlayer region [10]. A similar effect was observed previously on Pt(111) during the formation of a copper monolayer [11]. Bennewitz et al. observed such an increase on Au(111) upon Cu adsorption during potential cycling [12]. Obviously, this increase in friction is not a transient behaviour as we assumed from the above mentioned previous results involving cyclic voltammetry and potential steps across the peaks. In this context it is noteworthy that Hölzle and Kolb observed a nucleation and growth mechanism for peaks A2, B1 and B2, but not for A1 [31]. It is therefore not clear whether at the peak potential of A1/A2 the surface is covered by many small 2D nuclei or a high density of (freely diffusing) Cu adatoms; whereas the explanation of the increased friction due to many 2D islands involving many steps leading to an increased friction due to the Schwoebel barrier is straightforward, the explanation of the friction increase by adatoms would involve a more sophisticated model.

**Figure 4:**
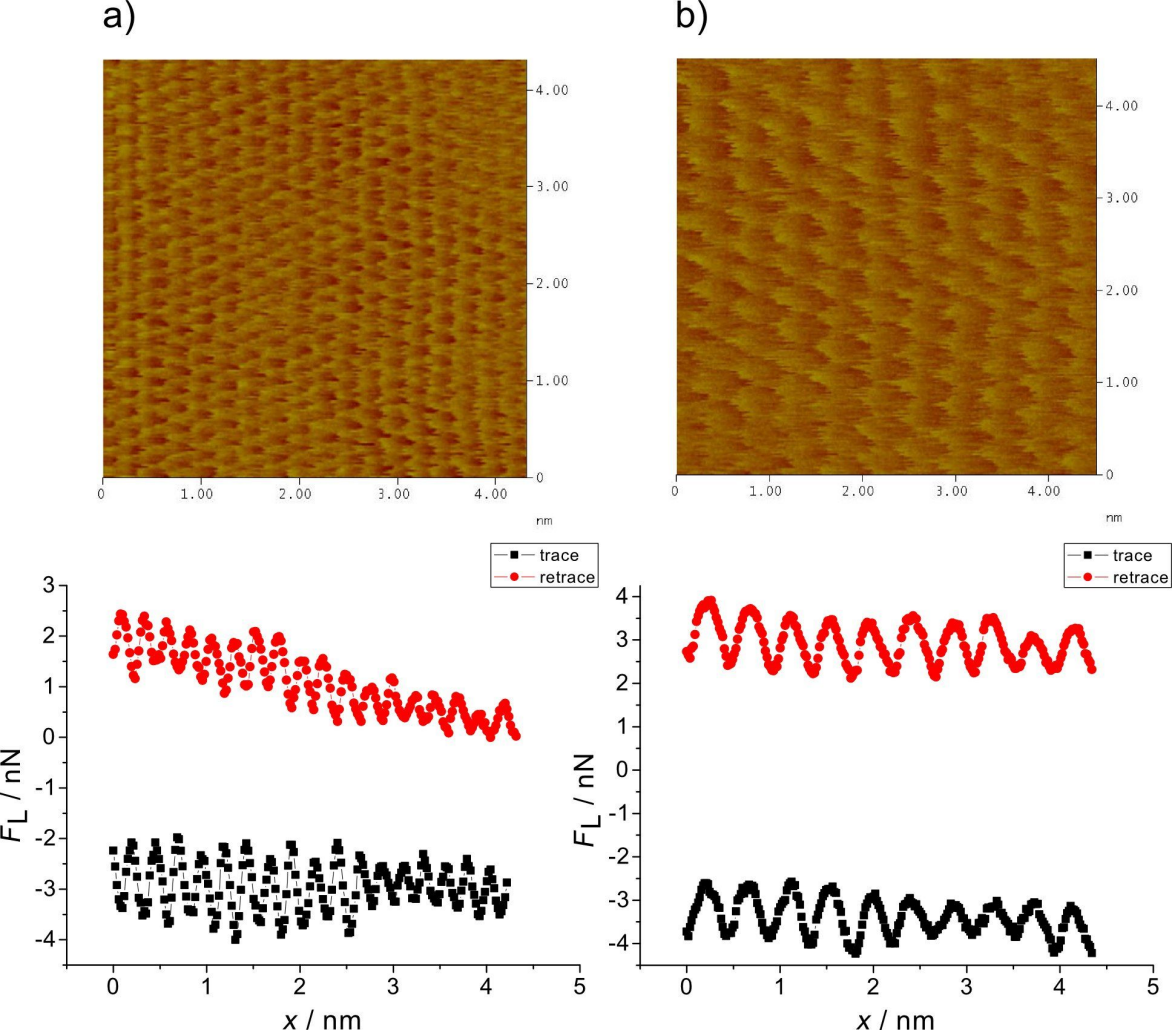
Stick–slip atomic resolution and cross section analysis for a) Au(111) at *E* = 270 mV; *F*_N_ = 80 nN; scan rate 106 nm/s; b) Cu 2/3, *E* = 100 mV; *F*_N_ = 45 nN; scan rate 92 nm/s.

Figure 4 shows atomically resolved images with high resolution of atoms for Au(111) (Figure 4a) and for Cu 2/3 (Figure 4b). The stick–slip periodicity on Au(111) (here at an angle of 30° to the lattice vector) equals 2.4 Å (error estimated from many images ±0.3 Å), which corresponds to √3/2 of the lattice vector. From that, the lattice vector is calculated to 2.71 Å. For the 2/3 copper layer the distance between two neighbouring potential minima from the stick–slip signal (now in the direction of the substrate lattice vector) equals 4.4 Å, which corresponds to 1.5 of the Au(111) periodicity, from which a lattice vector of 2.9 Å is calculated. Both values agree fairly well with the lattice vector of Au of 2.77 Å.

**Figure 5:**
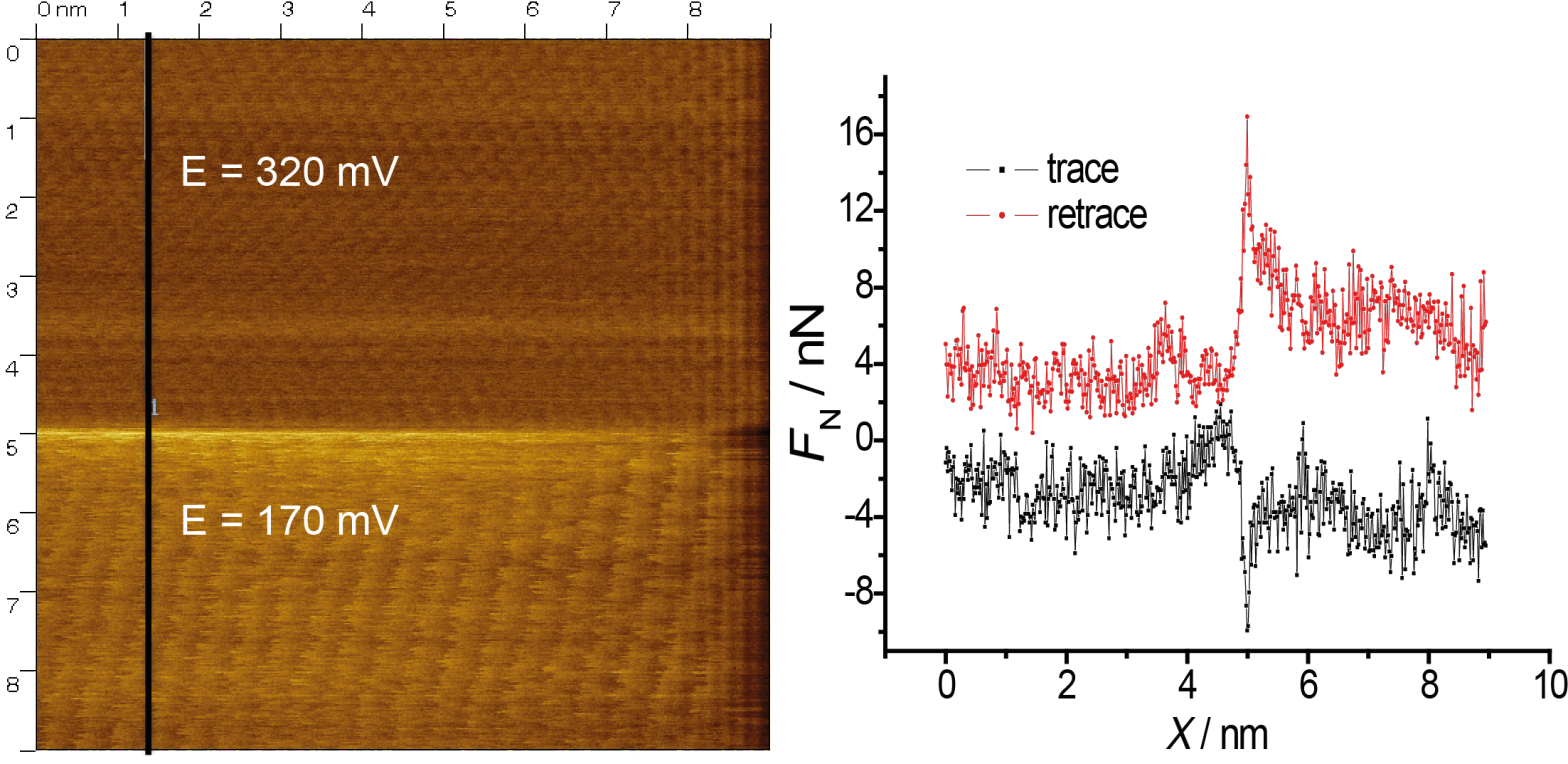
Stick–slip atomic resolution and cross section analysis for **a** potential jump from 320 mV (Au(111)/sulfate) to 170 mV (2/3 Cu adlayer), cross section along the vertical line as indicated; *F*_N_ = 45 nN; scan rate 275 nm/s.

The slope of the sawtooth stick–slip curve (section analysis below the figure) characterizes the effective lateral stiffness of the surface–tip contact. In our case it is 10 N/m and therefore much smaller than the lateral stiffness of the cantilever (190 N/m). The somewhat rounded shape might be due to a not completely commensurable tip–substrate contact [32]. Figure 5 shows the change of the structure during a potential step from the sulfate covered Au(111) surface to the Cu 2/3 adlayer. The increase in friction at the transition (cf. Figure 3b) is clearly seen.

**Figure 6:**
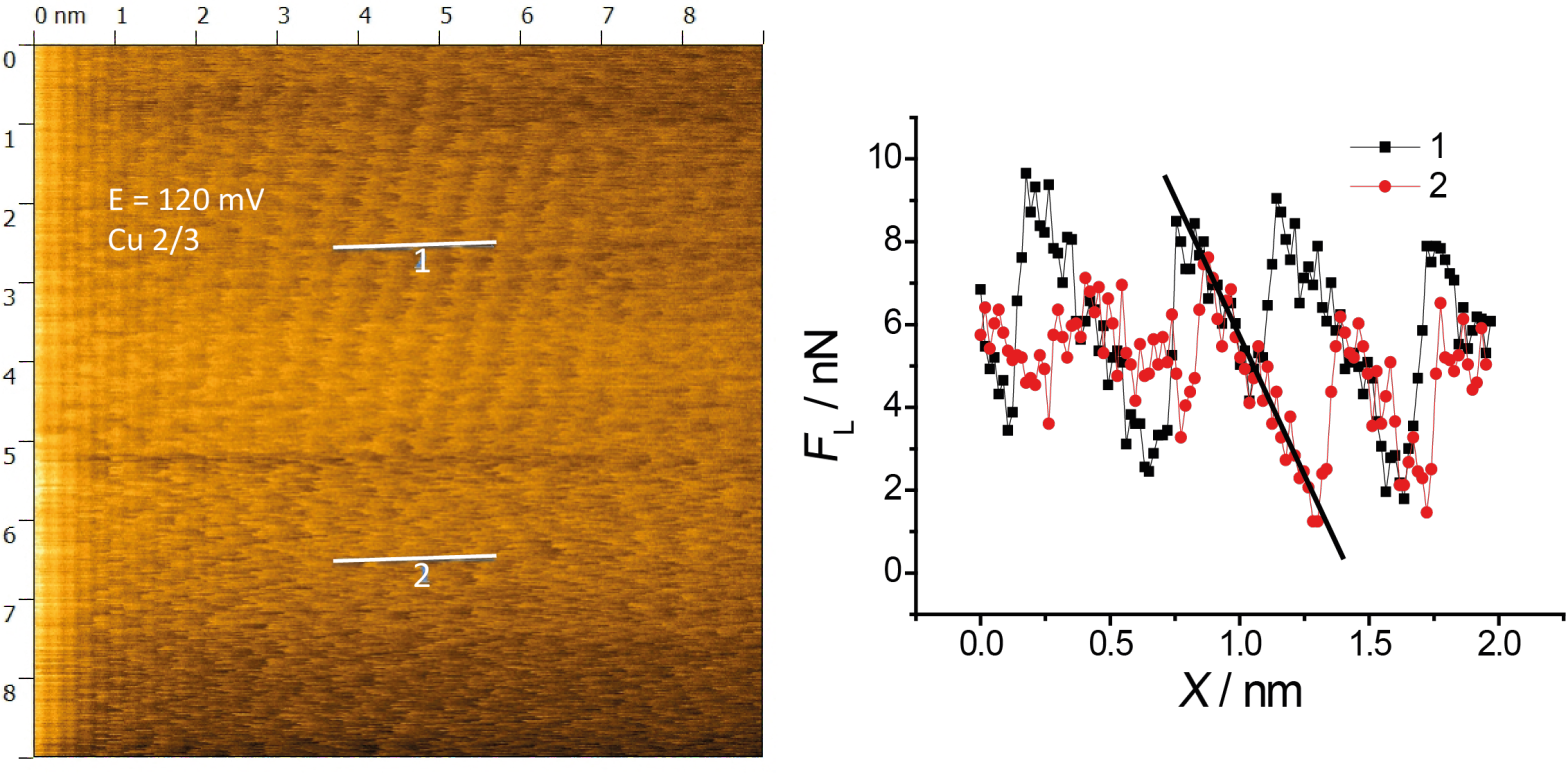
Stick–slip atomic resolution and cross section analysis for a potential jump from 0 mV (full Cu adlayer) to 120 mV (2/3 Cu adlayer), cross section along the two short horizontal lines *F*_N_ = 45 nN; scan rate 275 nm/s.

Figure 6 shows the friction force map during a change from the Cu monolayer to the 2/3 Cu layer. The slope of the stick curve is 12 N/m. In all cases it is between 10 and 12 N/m and independent of potential. It is important to mention that at all potentials the surface is very resistant to wear; even at high normal loads of about 250 nN, atomic resolution is always visible. At the pzc the quality of stick–slip resolution on gold is much better than at potentials of high sulfate coverage, in accordance with the results in [16].

**Figure 7:**
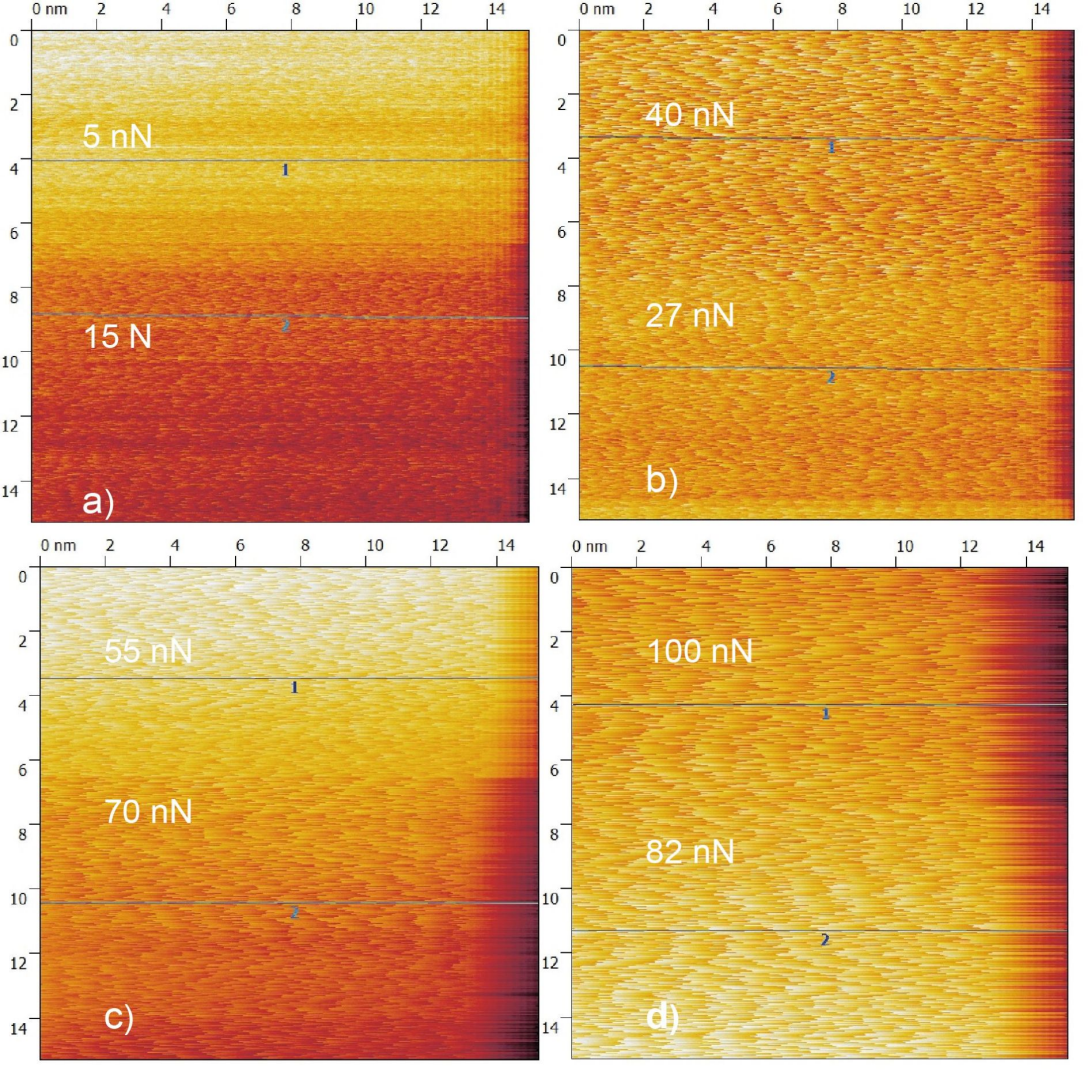
Copper monolayer friction images at *F*_N_ = a) 5, 15 nN; b) 27, 40 nN; c) 55, 70 nN and d) 82, 100 nN.

On the copper monolayer, stick–slip behaviour can be observed while atomic resolution (in y-direction) is completely absent in all applicable regimes of normal load. Figure 7 shows four friction force maps for eight normal loads at constant potential (*E* = 0 mV). At first sight the images appear irregular but the particular analysis of stick–slip behaviour shows astonishing results: With an increase of normal load the stick–slip periodicity increases.

**Figure 8:**
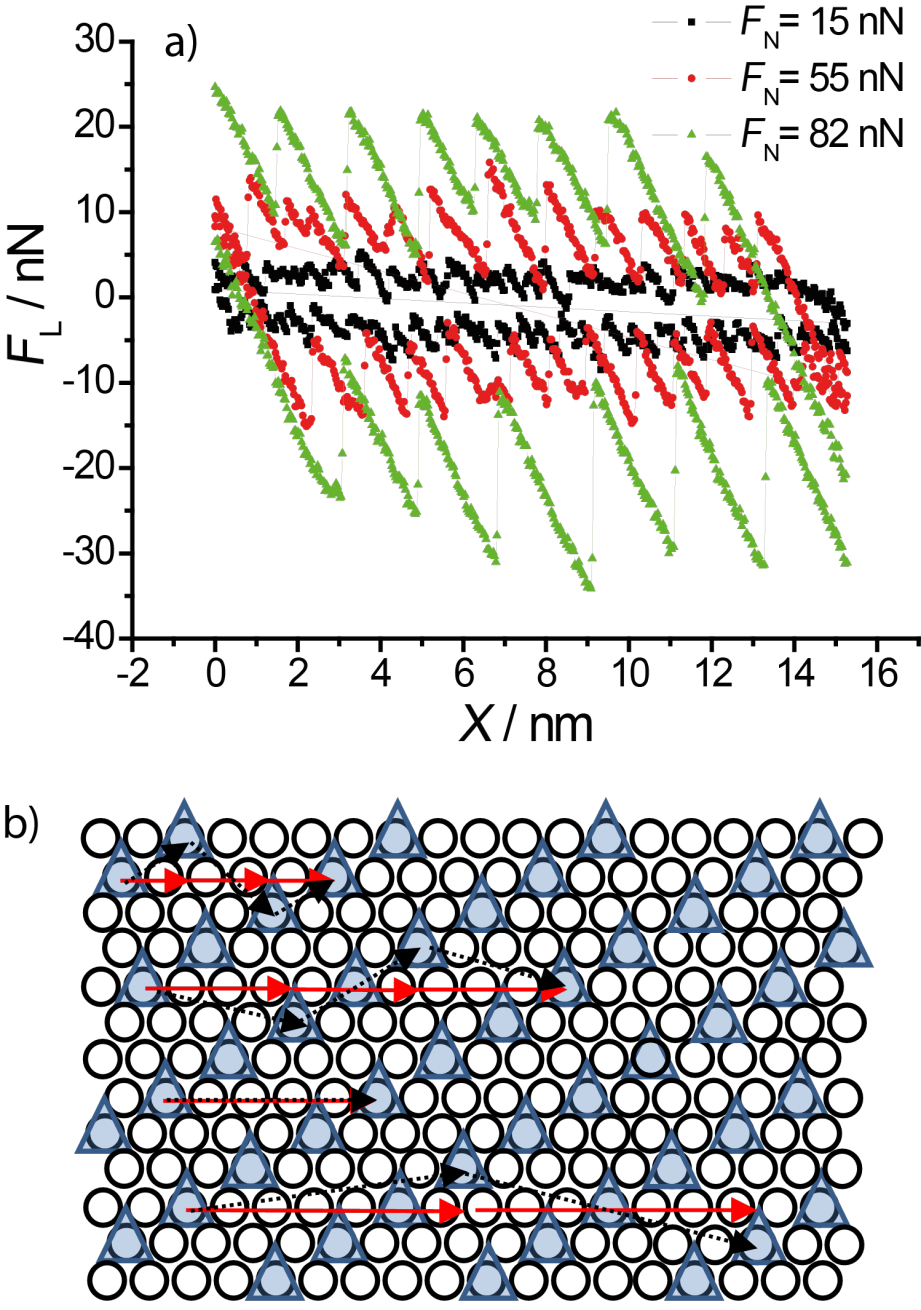
a) Typical cross sections from Figure 7 at *F*_N_ = 15, 55 and 82 nN. b) Schematic illustration of the √7 × √3 structure of sulfate anions (triangles) on a copper monolayer (circles): arrows (black, dotted) denote a possible jumping pathway for the AFM tip in a single, double, triple and quadruple slip. (Of course, triangles may as well reflect minimal energy positions between the sulfate ions.)

Figure 8a presents three typical cross sections from Figure 7. Typical slip distances are 4.6 Å or less for *F*_N_ ≈ 15 nN, 9.5–11 Å for 27 < F_N_ < 55 nN and 16.5 Å or more for 70 < *F*_N_ < 100 nN. This leads to the conclusion that at high normal loads, slips across multiple potential minima are observed. A similar effect was observed by Meyer and coworkers [33]: Upon an increase in normal load on a NaCl(001) surface a transition to multiple slip was found. According to [34], who predicted such transitions from theory for low damping conditions and also observed it on HOPG, this process is based on energy minimisation and caused by the increase in normal load.

A schematic model of the √7 × √3 sulfate structure on the Cu monolayer is presented in Figure 8b. Arrows represent a possible slip pathway for the different slip lengths. It is based on the crystal orientation as obtained from the Au(111) and Cu 2/3 structures. We assume that the tip apex is mobile enough in order to have a small variation in direction perpendicular to the tip motion. Black dotted lines on the Figure 8b show the path of tip apex while the red arrows represent the cantilever motion in scan direction. For a single jump, an average value of 0.46 nm (3 jumps within a distance of 5 lattice constants) is obtained, for a double jump 0.92 nm, for a triple jump 1.39 nm and for a quadruple jump 1.8 nm. These values agree fairly well with the experimental data.

**Figure 9:**
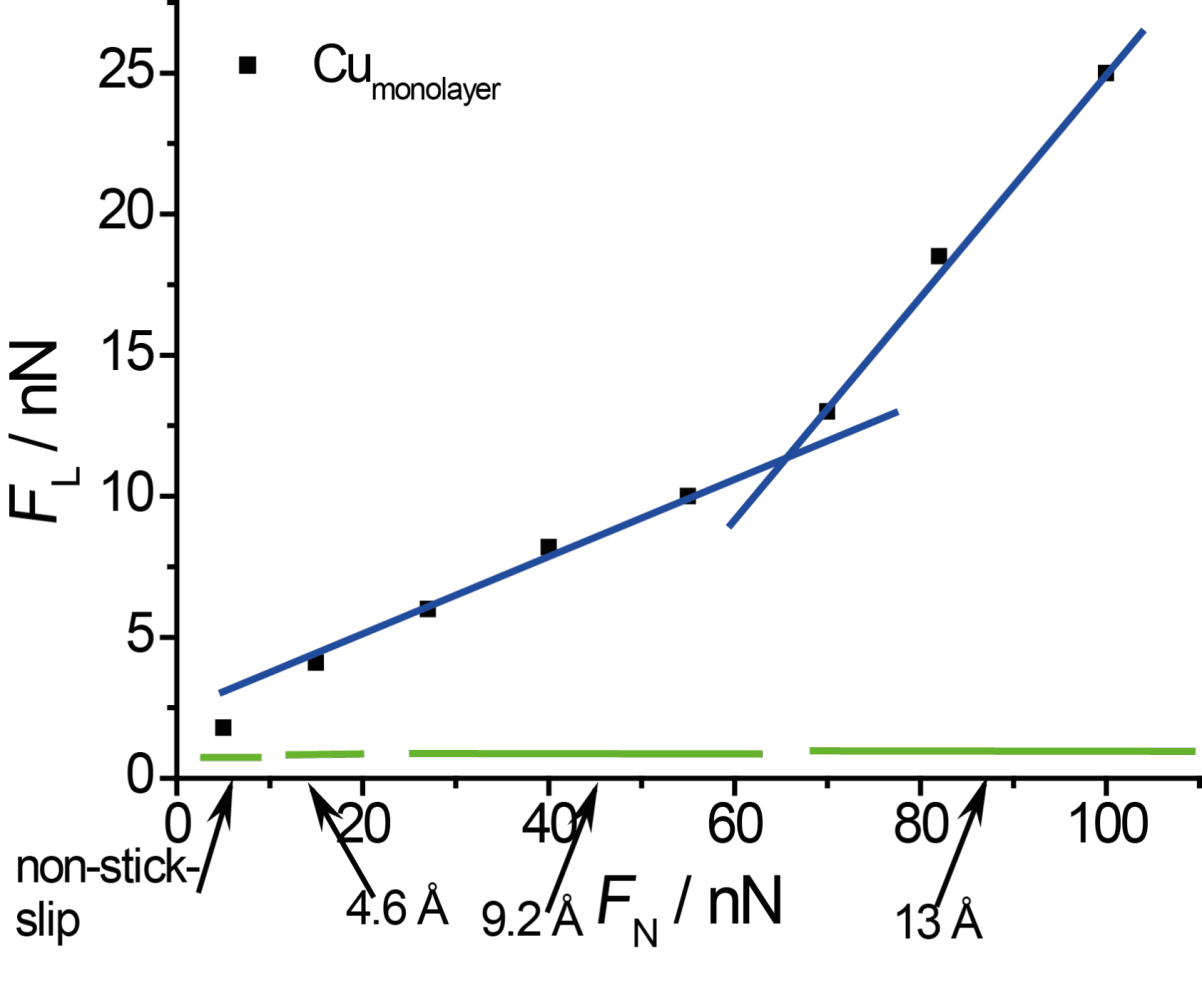
Friction force as a function of normal load (data, e.g., from Figure 8a).

A plot of the nano-scale friction force as a function of normal load on a copper monolayer is given in Figure 9. The friction coefficient changes from 0.12 to 0.5 at a normal load of approximately 70 nN. This change corresponds to change in friction coefficient presented on Figure 3a. This transition in friction coefficient goes along with a change in slip length, as indicated by the bars at the bottom. These friction data obtained from high resolution images agree well with those obtained from large scale images (cf. Figure 3a), although the transition at 15 nN is less visible than in Figure 3a because of the much lower density of data points.

**Figure 10:**
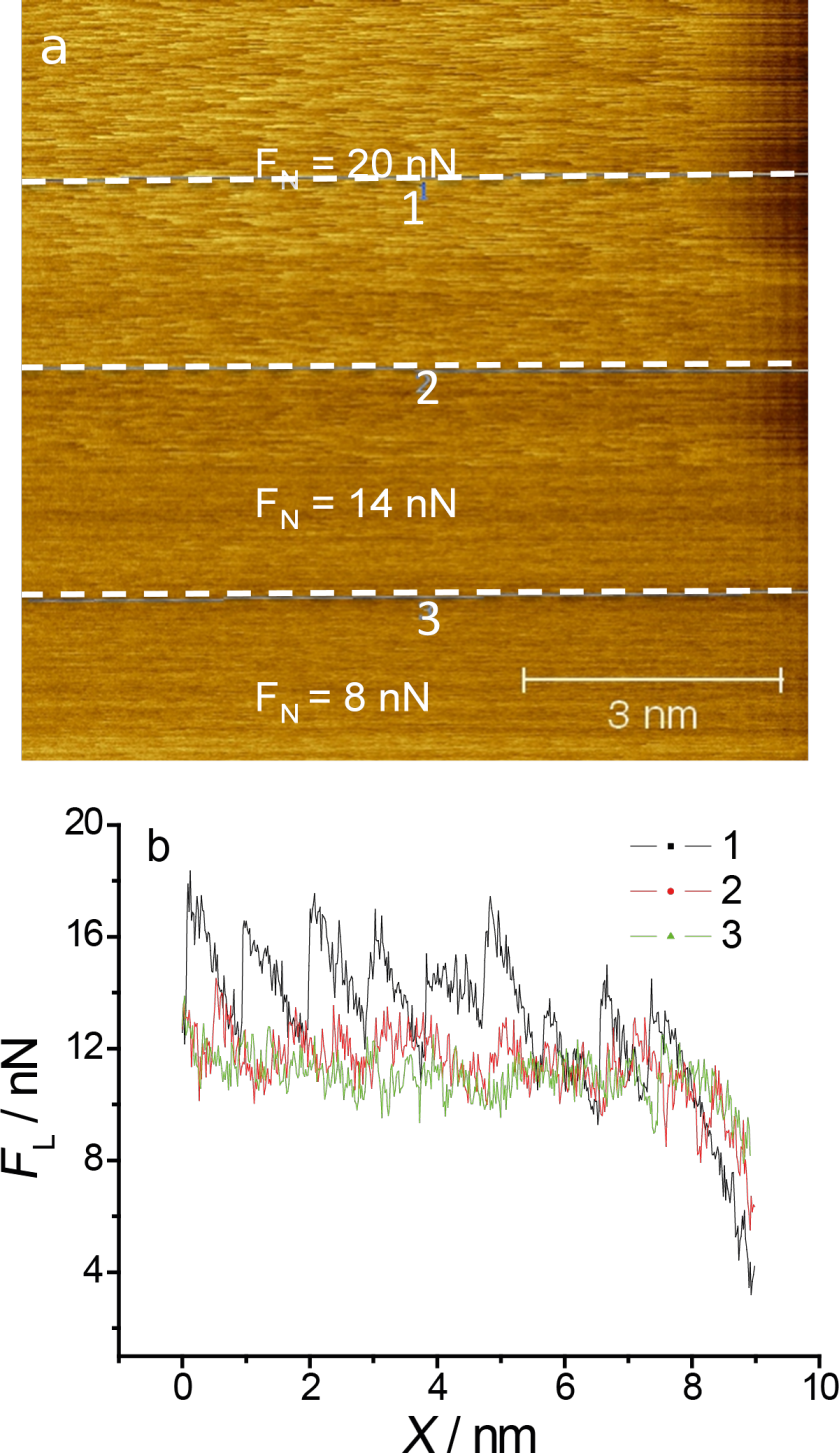
Transition from irregular to regular stick–slip on a copper monolayer a) AFM image at three different normal loads; b) cross sections along the lines from image a). *E* = 10 mV; scan rate 213 nm/s.

An important point is the vanishing of stick–slip behaviour at normal loads of less than 15 nN. In spite of non-stick–slip resolution the contact between tip and surface is preserved and energy dissipation is still observed. A transition to superlubricity would involve disappearing friction [35–36]. Since this is not the case here one might speculate about different contributions to friction, only one of which is disappearing with decreasing load. Figure 10 demonstrates the transition from non- to regular stick–slip on a Cu monolayer on Au(111). A similar effect is also observed at a 2/3 Cu monolayer and at the sulfate covered gold surface.

**Figure 11:**
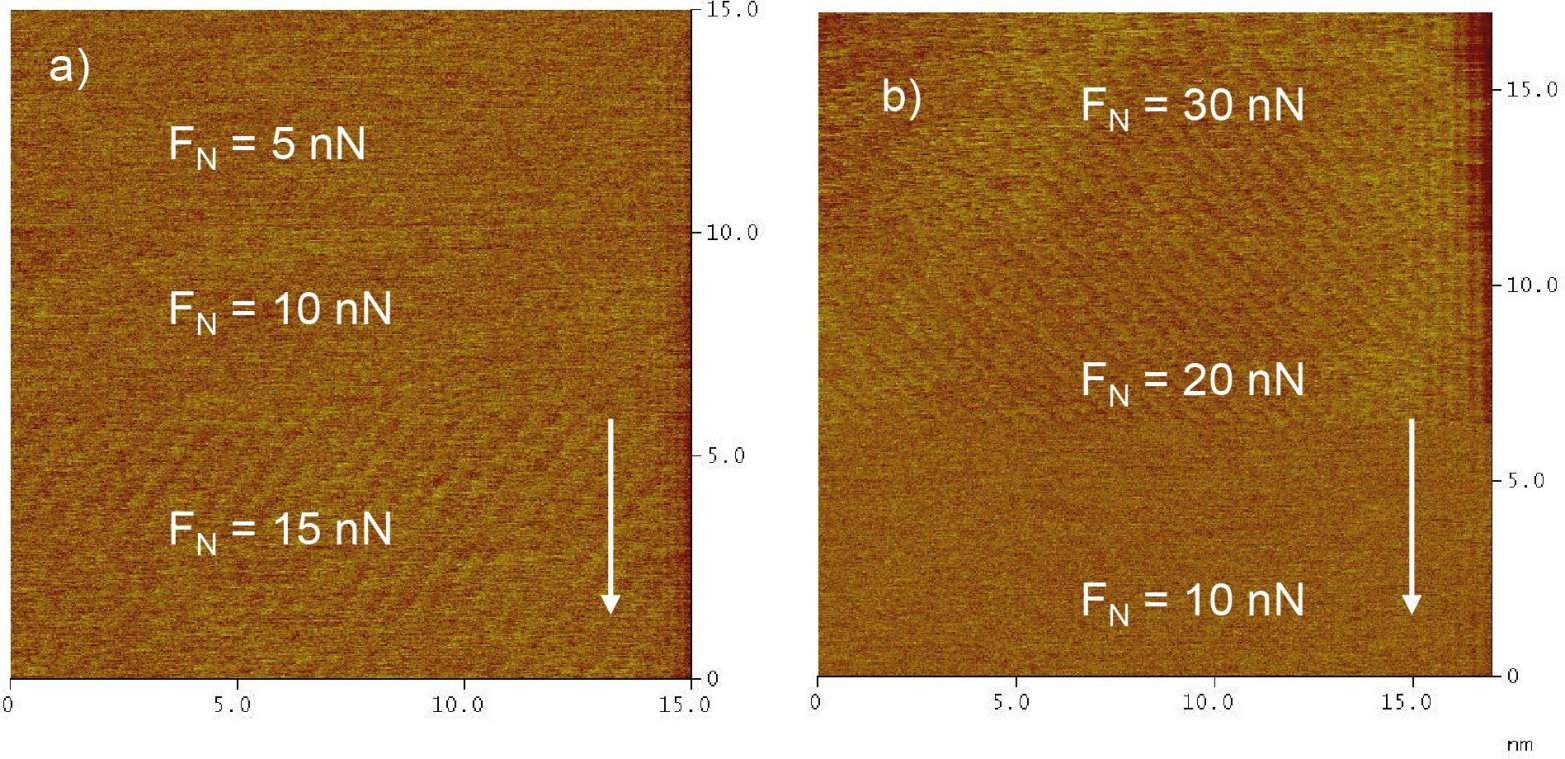
Transition between regular and irregular stick–slip on copper 2/3 during an increase (а) and a decrease (b) of normal load. *E* = 100 mV; scan rate 180 nm/s.

The transition between non-stick–slip and stick–slip on the copper sub-monolayer is shown in Figure 11. At normal loads above 10 nN regular atomic stick–slip without resolution in the slow scan direction is observed. Comparison of Figure 11a and b shows that the transition is reversible with regard to whether the normal load is increased or decreased. Good atomic resolution was only observed at higher normal loads (cf. Figure 4–6). Since stick slip disappears rather abruptly and reproducibly upon decreasing the normal load, we believe that this is a true physical effect and that stick slip does not just become indistinguishable from noise at lower normal loads.

**Figure 12:**
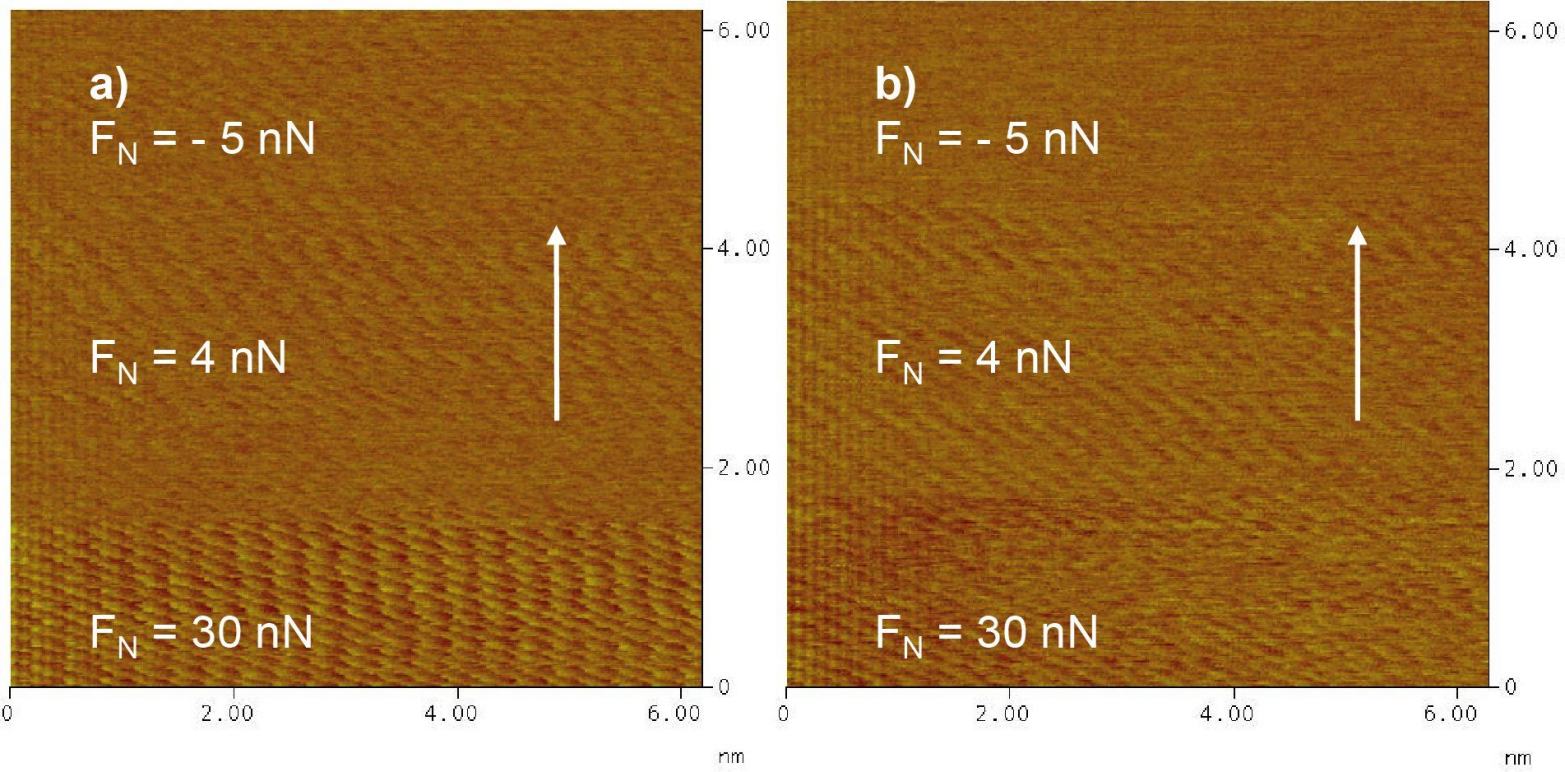
Friction images on Au(111). a) atomic resolution on Au(111) (no transition to non-stick–slip at this potential); *F*_N_ = −5, 4, 30 nN; *E* = 250 mV; scan rate 154 nm/s. (In this image, the apparent gradual change of the lattice direction at the beginning of the upward scan – lower part of the image – is an artefact probably due to the hysteresis of the scanner piezo.) b) Transition from non- to regular stick–slip behaviour at *F*_N_ = 4 nN; *E* = 420 mV; scan rate 154 nm/s.

Figure 12 shows friction force maps for two different potentials. At the pzc on clean gold (Figure 12a), stick–slip behaviour is present even at very small normal loads of nominally *F*_N_ = −5 nN. (The negative normal load is possible here because of adhesion, see below.) At normal loads below this value the contact between tip and surface is broken. On sulfate-covered gold at *E* = 420 mV (Figure 12b), a transition from non- to regular stick–slip behaviour is found for positive *F*_N_.

**Figure 13:**
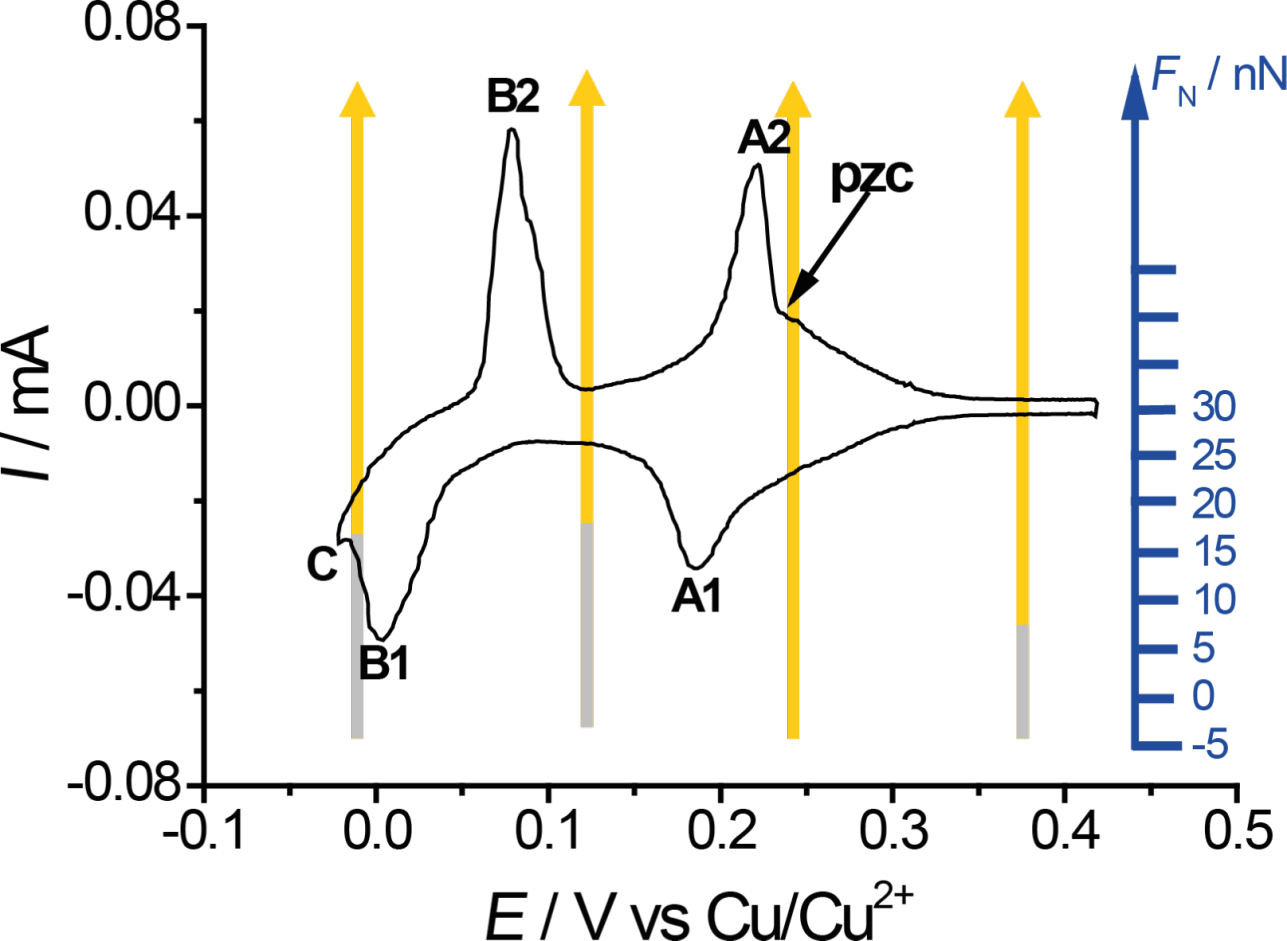
Cyclic voltammogram of Au(111) in 0.05 M H_2_SO_4_ + 4 × 10^−4^ M CuSO_4_ as shown in Figure 1. Normal force regions of different behaviours are inset as arrows. Orange: atomic-resolution stick–slip; grey: non-stick–slip behaviour.

For Figure 13, all experimentally observed transition forces were collected and correlated to the applied potential. Orange arrows mark the force region where stick–slip is observed. The grey parts of the arrows correspond to regions of non-stick–slip. Figure 13 shows that for both the sub- and monolayer of copper the transition to regular stick–slip starts at the same normal load of about 15 nN. At this same normal load we observed a transition in friction coefficient on the microscale for Cu covered gold (Figure 3). As mentioned before, sulfate is adsorbed to the copper layer for both the 2/3- and the monolayer. Also positive of the pzc, where only sulfate is adsorbed, such a transisiton is observed, but not at the pzc, where atomic stick–slip is observed even at low normal loads. These effects lead to the conclusion that the transition is strongly dependent on adsorption. The onset of stick–slip may be caused by a tip penetration through the electrochemical double layer, which effectively is not existent at the pzc. Possibly at potentials other than the pzc, the tip is scanning above or in a viscous layer of water above the double layer at low loads.

**Figure 14:**
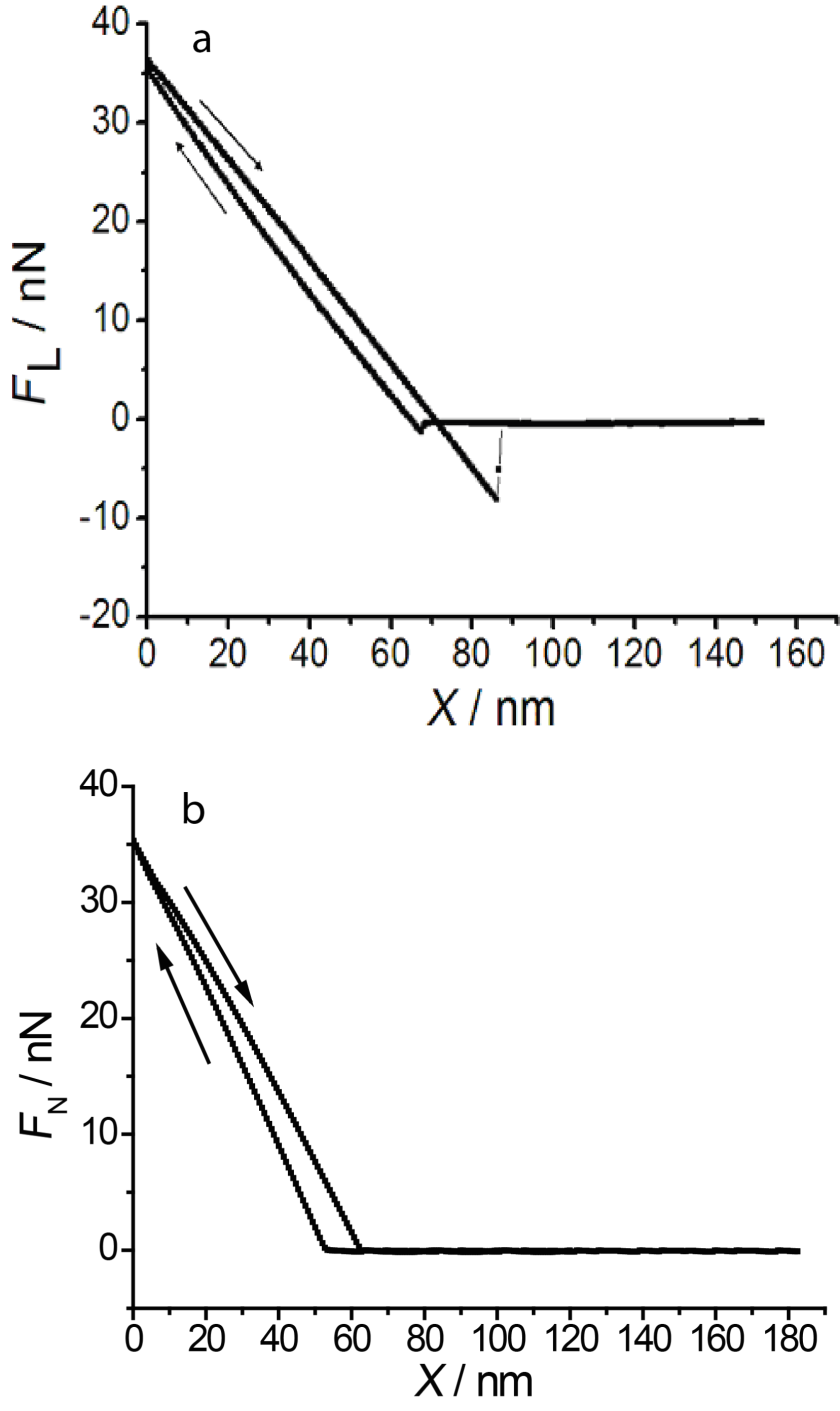
Force–distance curves at potentials of a) 220 mV, b) 150 mV. A large snap-off force due to adhesion is only found for potentials positive of the pzc.

Figure 14 shows force–distance curves at different potentials. A curve showing strong adhesion (Figure 14a) is observed in the potential region positive of and at the pzc where the gold surface is free of adsorbates or covered with a sulfate layer; the value of the adhesion is independent of the potential. For all potentials negative of the pzc including the copper monolayer the adhesion disappears (Figure 14b). This could be related to a different orientation of water molecules in the double layer and a possible formation of hydrogen bonds to the OH groups of the surface oxide of the Si tip. (In principle, these tip approach curves should also reveal the above mentioned penetration of the tip into the double layer. Unfortunately, at present the resolution is not sufficient to reveal the corresponding small vertical tip displacement of about 0.2 nm corresponding to the atomic diameter of, e.g., a Cu atom. Corresponding work is in progress.)

An origin of the attractive interaction positive of the pzc is also the electrostatic interaction between the positively (protonated) charged tip and the negatively charged surface (caused by the specific adsorption of sulfate). The irreversibility may also be caused by a mutual penetration of the two double layers (that of the surface and that of the tip) into each other and an irreversibility of the building up of the original double layer upon withdrawal of the tip. At low potentials, due to the Cu adlayer, the double layer of the substrate could be much more rigid. This could also explain the non-stick–slip behaviour at Cu-covered surfaces: at low normal loads, tip and surface are separated by their own double layers and atomic resolution is not obtained.

## Conclusion

Friction forces on Au(111) covered by a copper monolayer in sulfuric acid solution show several transitions in friction coefficient which are also related to a transition to multiple-slip. The stick length corresponds to multiples of the distance of adsorbed sulfate. Stick–slip behaviour for both the copper monolayer and 2/3 Cu coverage can be observed at normal loads above *F*_N_ = 15 nN. (This value is certainly dependent on the tip radius, which, as mentioned above, is around 100 nm.) At the same normal load a change in friction coefficient on the microscale can be found. This may suggest that at this normal load, the tip penetrates an outer water layer of the electrical double layer. Stick–slip resolution at potentials close to the pzc can be observed at all normal forces investigated.

Friction is minimal at the potential of zero charge, suggesting again the role of adsorbates for frictional energy dissipation. On the other hand, friction is particularly large when the adlayer is disordered at the potential of a phase transition, as shown here for the formation of the √3 × √3 adlayer. Whether these phenomena can be generalized has to be elucidated in further studies.
